# Application of neurophysiological monitoring during tethered cord release in children

**DOI:** 10.1007/s00381-024-06483-9

**Published:** 2024-06-08

**Authors:** Junjun Guo, Xianlan Zheng, Hongyao Leng, Qiao Shen, Jialin Pu

**Affiliations:** 1 Department of Neurosurgery, Children’s Hospital of Chongqing Medical University, National Clinical Research Center for Child Health and Disorders, Ministry of Education Key Laboratory of Child Development and Disorders, Chongqing Key Laboratory of Structural Birth Defect and Reconstruction, Chongqing, 400000 China; 2 Department of Nursing, Children’s Hospital of Chongqing Medical University, Chongqing, 400000 China

**Keywords:** Intraoperative neuroelectrophysiological monitoring, Neural tube defects, Children

## Abstract

**Objective:**

The objective of this study was to explore the effect of intraoperative neurophysiological monitoring (IONM) on tethered spinal cord release in children.

**Methods:**

The clinical data of 454 children with tethered cord syndrome who underwent surgery for tethered cord release were retrospectively analyzed. The children were divided into two groups: the non-IONM group and the IONM group. SPSS 26.0 software was used for statistical analysis. The evaluation indices included the effective rate and incidence of new neurological dysfunction.

**Results:**

The short-term results showed that the effective rate of the non-IONM group was 14.8%, while that of the IONM group was 15.2%. Additionally, the incidence of new neurological dysfunction was 7.8% in the non-IONM group and 5.6% in the IONM group. However, there was no significant difference between the two groups (*P* > 0.05). The medium- to long-term follow-up had significant difference (*P* < 0.05), the response rate was 32.1% in the IONM group and 23.7% in the non-IONM group, and deterioration rates regarding neurological dysfunction were 3.3% in the IONM group and 8.5% in the non-IONM group.

**Conclusion:**

This study revealed that the use of IONM does not significantly improve the short-term treatment effect of patients undergoing surgery for tethered cord release or reduce the short-term incidence of postoperative new neurological dysfunction. However, the medium- to long-term prognoses of patients in the IONM group were better than those of patients in the non-IONM group.

## Introduction

Tethered cord syndrome (TCS) is a congenital disease involving neural tube defects, accounting for approximately 20.2% of congenital malformations of the central nervous system in children, ranking third [1]. TCS in children often associated with local skin abnormalities, bladder/rectal dysfunction, lower extremity dysfunction and/or deformities, and pain due to TCS is rare [2, 3]. Surgery for tethered cord release is the gold standard treatment for TCS [4], but nerves may be injured during surgery, leading to neurological dysfunction complications. Intraoperative neurophysiological monitoring (IONM) can assist in detecting nerve damage and preventing accidental nerve injury. The effective rate of IONM in children undergoing surgery for TCS has been reported to vary from 63.4 to 98.6% [5–7], and its application effect is controversial. A study of the efficacy of IONM in children undergoing surgery for TCS was conducted to clarify the application effect of the procedure.

## Methods

### Data collection

Using electronic medical records, hospital information systems, and a self-designed Excel data collection form, data were collected from patients admitted between July 1, 2011, and September 30, 2021, who met the inclusion and exclusion criteria. The inclusion criteria included patients who were diagnosed with TCS and who underwent surgery for tethered cord release for the first time. The exclusion criteria included patients who were not undergoing surgical treatment for tethered cord release, who had a central infection, and who had incomplete information. The collected data included the following: basic patient data, such as sex, age, disease course, length of hospital stay, and diagnostic classification; data on preoperative conditions, including symptoms, positive signs, and complications; data on intraoperative conditions, including operation time, and intraoperative release degree; and data on postoperative conditions, including postoperative positive signs and symptoms and postoperative complications. Ultimately, 454 patients were identified, and data verification was performed by two nurses who had worked in this specialty for more than 5 years and were skilled in data collection and office software operation. In 2015, we introduced neurophysiological monitoring, which was first used on May 1; therefore, patients were grouped according to changes in clinical practice. No IONM was performed from July 1, 2011, to April 30, 2015. Patients treated in this period were included in the non-IONM group, and patients treated from May 1, 2015, to September 30, 2021, were included in the IONM group.

### Neurosurgeons

All patients were operated on by paediatric neurosurgeons (*n* = 4) with a mean age of 44 years, a master’s degree or above, and a professional title of attending physician or above, including 1 attending physician, 1 associate chief physician, and 2 chief physicians, who had the same experience in performing surgery for TCS. There were no differences (*P* > 0.05) in postoperative prognosis (*P* = 0.441), postoperative complications (*P* = 0.453), the degree of tether release (*P* = 0.421), or intraoperative blood loss (*P* = 0.092).

### Monitoring methods

An American CADWELL intraoperative–evoked potential instrument and a cascade intraoperative monitoring system were utilized for multimodal monitoring. The monitoring methods included transcranial electrical stimulation–muscle motor–evoked potentials (Tc-MEPs), somatosensory evoked potentials (SSEPs), free tracing electromyography (F-EMG), and triggered electromyography (T-EMG). According to the International Electroencephalogram 10/20 system standard, head electrodes were placed, Tc-MEPs were monitored to evaluate the motor pathway, and the motor cortex was stimulated by constant piezoelectric stimulation through scalp C1 and C2, with channels set at 9. EMG responses were recorded from the bilateral quadriceps femoris, tibialis anterior, gastrocnemius, abductor muscle, and anal sphincter. SSEPs were used to monitor the sensory pathway in the dorsal spinal cord, and evoked potentials were recorded from the scalp CZ through constant current stimulation of the posterior tibial nerve behind the peripheral medial malleolus. Tc-MEP and SSEP baselines were established before the proceeding incision was made, with an intraoperative test frequency of approximately 20–30 min, and the test frequency was increased according to the surgical conditions. SEMG was used to monitor intraoperative conditions in real time, and muscle action potentials were recorded directly from the electrodes of 9 channels. The selective use of T-EMG, that is, intraoperatively by doctors according to the surgical situation, if necessary, involves the use of a stimulator to directly stimulate suspicious tissues or nerves and monitor whether there is a corresponding EMG response.

### Evaluation indicators

The therapeutic effect was evaluated according to the changes in the Hoffman functional classification before and after the operation. A cure/significant improvement was defined as a Hoffman grade increase by ≥ 2 grades or improved dysfunction in urination, defecation, lower limb muscle strength, and sensation after the operation compared with before the operation; improvement as defined as a Hoffman grade increase by 1 grade after the operation compared with before the operation and one or more improvements in urination, defecation function, and neuropathy; no change/no progression was defined as postoperative neurological symptoms that were consistent with preoperative neurological symptoms; and deterioration was defined as neurological dysfunction symptoms after the operation that were worse than those before the operation or the occurrence of new neurological dysfunction. The effective rate was used as the main indicator to evaluate the treatment effect and was calculated as (cured/significantly improved patients + improved patients)/number of patients operated on × 100%. Additionally, the incidence of postoperative new neurological dysfunction was used as a secondary indicator to evaluate treatment safety. The incidence of postoperative new neurological dysfunction was calculated as the number of patients with postoperative new neurological dysfunction/number of patients who underwent surgery × 100%.

### Statistical analysis

SPSS 26.0 software was used for statistical analysis. Enumeration data are reported descriptively by frequencies and percentages, and differences in basic data between the groups were balanced using propensity score matching. Pearson’s chi-square test and Fisher’s exact test were used to compare differences in basic data and the rates between the two groups.

## Results

Overall, 454 children aged 0.3 months to 178 months, with a mean age of 21.0 months, were included in this study; 215 were male and 239 were female, with a disease duration of 0.1 months to 156 months and a mean duration of 17.4 months. The longest duration of short-term follow-up was 72 days, with a mean of 11.95 days. Preoperative symptoms and signs (see Table 1) and sacrococcygeal skin abnormalities were observed in 93.83% of the patients, and bladder/rectal dysfunction was observed in 21.81% of the patients; 128 patients had malformations, of which foot varus/equinus (6.83%), congenital heart disease (6.61%), urinary system malformations and/or renal dysfunction, hydronephrosis (6.17%) (see Table 2), and lipoma and myelomeningocele classifications were more common (see Table 3). In addition, the overall treatment response rate in this group of patients was 15.9%, and the incidence of new postoperative neurological dysfunction was 6.6%. A comparison of the basic characteristics of the groups revealed differences. The sex distribution varied between the non-IONM group and the IONM group, with a greater proportion of males in the non-IONM group before May 2015 and a slightly greater proportion of females in the IONM group from May 2015 onwards. This shift may be linked to the declining sex ratio at birth in recent years [8]. Additionally, differences in blood type and payment method may also be attributed to objective factors, while no significant disparities were observed in other basic conditions (see Table 4 for a comparison of treatment outcomes). As shown in Table 4, there were differences in the basic data between the two groups, so we used propensity score matching to balance the differences between each factor (sex, age, ethnicity, source (local, migrant), disease duration, total hospital stay, subtype). Before the comparison, 365 patients were successfully matched, including 250 patients in the IONM group and 115 patients in the non-IONM group. Additionally, all the matched variables were balanced after matching (there were no statistically significant differences in any of the factors between the two groups, *P* > 0.05), and the matching effect improved. The chi-square test was then used, and the analysis revealed that the effective rate was 15.2% in the IONM group and that in the non-IONM group was 14.8% (see Table 5). From Table 5 we can also see that there was no statistically significant difference between the two groups, and there was no statistically significant difference in the incidence of the new neurological dysfunction between the IONM group (5.6%) and the non-IONM group (7.8%). The incidence of non-neurological dysfunction complications was 36.0% in the IONM group and 37.4% in the non-IONM group (*P* > 0.05).

**Table 1:** Preoperative related symptoms and signs (*n* = 454)

Preoperative symptoms and signs	Number of cases (*n*)	Percentage (%)
Without symptoms	13	2.86
Abnormal sacrococcygeal skin	426	93.83
Muscular dysfunction of lower extremity	29	6.39
Bladder/rectal dysfunction	99	21.81
Lower limb deformity	39	8.59
Pain/sensory disturbances	6	1.32

**Table 2:** Preoperative congenital malformation (*n* = 454)

Combined congenital malformations	Number of cases (*n*)	Percentage (%)
Without	327	72.03
No anorectal/rectal stenosis	24	5.29
Hydrocephalus	5	1.10
Foot varus/equinus congenital	31	6.83
Congenital heart disease	30	6.61
• Scoliosis/dorsi-biconvex	11	2.42
Hypospadias/cryptorchidism/ectopic kidney and/or renal dysfunction	28	6.17
Other congenital malformations (polydactyly, chairi malformation, etc.)	25	5.51

**Table 3:** TCS classification of patients (*n* = 454)

Typed	Number of cases (*n*)	Percentage (%)
Filum terminale	74	16.30
Sacrococcygeal lipoma	195	42.95
Myelomeningocele	86	18.94
Mixed type	85	18.72
Others	14	3.08

**Table 4:** Analysis of basic data of IONM group and non-IONM group (*n* = 454)

Project	Team	IONM group		non-IONM group		*χ*²	*P*
		Number of cases (*n*)	Percentage (%)	Number of cases (*n*)	Percentage (%)		
Gender	Male	145	43.15	70	59.32	9.156	0.002
	Female	191	56.85	48	40.68		
Age	Infant group	284	84.52	92	77.97	2.639	0.104
	School age group	52	15.48	26	22.03		
Ethnic	Han	294	87.50	108	91.53	1.395	0.238
	Others	42	12.50	10	8.47		
Patient source	Migrant	198	58.93	67	56.78	0.166	0.684
	Local	138	41.07	51	43.22		
Duration of disease (months)	≤ 36	301	89.58	93	78.81	8.832	0.003
	>36	35	10.42	25	21.19		
Preoperative hospital stay (days)	≤ 3	285	84.82	100	84.75	0.178	0.936
	3.1~7	42	12.50	14	11.86		
	>7	9	2.67	4	3.39		
Postoperative hospital stay (days)	≤ 7	30	8.93	6	5.08	3.161	0.411
	7.1~14	253	75.30	89	75.42		
	14.1~30	47	13.99	21	17.80		
	>30	6	1.79	1	0.85		
Total days in hospital (days)	≤ 7	7	2.08	0	0	5.082	0.150
	7.1~14	202	60.12	66	55.93		
	14.1~30	114	33.93	50	42.37		
	>30	13	3.87	2	1.69

**Table 5:** Comparison of treatment effect between IONM group and non-IONM group (*n* = 365)

Item	Group	IONM group		non-IONM group		*χ*²	*P*
		Number of cases (*n*)	Percentage (%)	Number of cases (*n*)	Percentage (%)		
Treatment effect	Effective	38	15.2	17	14.8	0.149	0.928
	No change	195	78.0	89	77.4		
	Ineffective	17	6.8	9	7.8		
New neurological dysfunction		14	5.6	9	7.8	0.661	0.416
Non-neurologic dysfunction complication		90	36.0	43	37.4	0.660	0.798

## Discussion

### IONM does not significantly improve short-term prognosis after surgery for TCS in children

The results showed that the short-term effective rate of the IONM group was slightly greater than that of the non-IONM group, but there was no significant difference in the effective rate between the two groups, so the use of IONM during surgery for TCS had no significant effect on the therapeutic effect. It has also been shown that although IONM does not significantly improve short-term prognosis after surgery for TCS, the use of IONM is significant for neurological improvement in long-term follow-up after surgery [9–11]. This was also the case in this study; the medium- to long-term follow-up durations ranged from 29 to 1727 days, and the median follow-up duration was 502 days. The groups with the medium- to long-term follow-up had significantly different (*P* = 0.026, *P* < 0.05) response rates (32.1% in the IONM group and 23.7% in the non-IONM group) and deterioration rates regarding neurological dysfunction (3.3% in the IONM group and 8.5% in the non-IONM group), so the medium- to long-term prognoses of patients in the IONM group were better than those of patients in the non-IONM group. Patients whose conditions deteriorated during long-term follow-up mainly presented with abnormal urination due to bladder dysfunction, as well as lower limb motor dysfunction associated with clubfoot varus, and patients were mainly treated in nephrology or urology, orthopaedics, and rehabilitation departments. In clinical practice, surgery for TCS is complex, with a high incidence of postoperative complications and poor overall outcomes. The surgical results are largely influenced by the complexity of the anatomy, preoperative clinical manifestations, and the rate of complete intraoperative release [11]. Long-term follow-up is more objective and realistic for neurological evaluation in patients with lipomatous TCS [12]. High-quality studies are needed to demonstrate whether functional improvement during long-term follow-up is significantly associated with the use of IONM. Furthermore, the high response rate (ranging from 63.4 to 98.6%) with the use of IONM for TCS patients reported in previous studies may be attributed to variations in evaluation indicators and follow-up cycles. Moreover, a report on the intraoperative use of IONM in cervical spondylotic myelopathy patients indicated that IONM increased the surgical preparation time and cost, but because false-positive alarms had a negative impact and had no significant benefit for patients undergoing surgery [13], similar reports have not been made in patients with TCS, and further studies are needed.

### IONM did not reduce the incidence of new neurological dysfunction after surgery for TCS in the short term

The incidence of new neurological dysfunction after surgery for TCS in the IONM group (5.6%) was slightly lower than that in the non-IONM group (7.8%), but the difference was not statistically significant (*P* > 0.05). Although the incidence of new neurological dysfunction in the IONM group in the present study was 5.6%, which was not significantly different from that in the non-IONM group, it was still lower than the currently reported rate of 9.18% [11]. In practice, the slightly greater incidence of nonneurological dysfunction complications (including incision infection, cerebrospinal fluid leakage, and urinary tract infection) may be caused by more complex disease conditions such as the lipomatous and mixed types, more difficult intraoperative release, and a greater incidence of postoperative complications [14].

Moreover, in this study, the more complex subtypes accounted for a relatively large proportion of patients in the two groups. In the IONM group, 154 patients (45.8%) had the lipomatous type, while in the non-IONM group, 41 patients (34.7%) had the lipomatous type. The incidence of nonneurological complications in the IONM group was lower than that in the non-IONM group, but the difference was not significant. The incidence of complications was 36.0% in the IONM group and 37.4% in the non-IONM group. In this study, the incidence of incisional infection and cerebrospinal fluid leakage was greater in the non-IONM group than in the IONM group, which may be related to poor nursing techniques regarding the initial incision and the dural suture technique. With the improvement in the dural suture technique and nursing technique by surgeons, the incidence decreased to some extent in the IONM group. Additionally, studies have confirmed that early warnings with the use of IONM during spinal cord surgery may be more sensitive, specific, and effective in high-risk groups than in low-risk groups [15]. However, because high-risk patients are more likely to develop severe dysfunction, multimodal IONM monitoring with distal multichannel monitoring is optimal [16]. The application of multimodal IONM in patients with lipomatous TCS can assist in the identification of normal neural tissue, thereby achieving maximum resection of lipomas while protecting neural tissue during surgery [17]. In addition, the signals of and responses to IONM vary according to the age of the child, and the type of surgery and the nerve conduction pathways that may be at risk determine the IONM pattern type used [18]. Although the use of IONM can protect spinal cord function, prevent neurological impairment, and reduce the occurrence of postoperative complications [8], IONM is susceptible to interference by nonsurgical factors such as body temperature, blood pressure, anaesthesia, and surgical technique [19]. This condition cannot be ignored and may also be responsible for intraoperative nerve injury or undetected nerve traction; therefore, it is very important to ensure the effectiveness of IONM from many aspects, considering anaesthesia, surgery, technology, and population factors.

### Weighing the advantages and disadvantages of IONM in paediatric TCS patients

Intraoperative IONM helps in the localization of neural structures and assists surgeons in preserving neurological function as much as possible [18]. Its role cannot be ignored, and free tracing electromyography monitoring can also reveal the interference of surgical procedures with nerves in real time [20]. In addition, the bulbocavernosus reflex (BCR) also has predictive value for postoperative voiding function in patients with lipomatous TCS [21]. Although most studies have shown that IONM provides an additional layer of assurance for patients and that its application is safe and effective, some studies on the use of IONM in patients with cervical spondylosis have indicated that it does not result in a lower risk of neurological dysfunction or loss but rather increases both time and hospital costs [13, 22]. Therefore, in the current medical environment and with ongoing medical development, the focus of continued research is how to use IONM more reasonably and effectively rather than as a routine practice to lower medical costs, maximize patient safety, and promote early postoperative rehabilitation.

## Summary

The use of IONM during cord release surgery in patients with TCS improves long-term outcomes but does not improve short-term treatment outcomes in children, nor does it reduce short-term new neurological dysfunction after surgery for TCS. The postoperative outcomes of patients with TCS may be more related to the disease itself, the degree of neurological dysfunction, surgical methods, and the complexity of classification. The application of IONM should be individualized, accounting for the clinical experience and judgement of doctors, as well as comprehensively considering MRI data, patient symptoms, and other factors. Multimodal IONM is still necessary for patients with severe preoperative symptoms and complex classifications. However, patients with asymptomatic or less complex TCS may benefit from a reduction in the monitoring mode and channels. Therefore, IONM executors should closely communicate and cooperate with surgeons to determine the indications for different disease states based on the characteristics of individual patients [23]. This approach is expected to result in a more scientific, rational, and cost-effective IONM regimen that provides greater benefits to paediatric patients. This was a retrospective study, so it has several limitations. At present, only the patient outcome was selected as an evaluation indicator, while the effect of the surgical process or surgeon and the predictive effect of the IONM data were not used for evaluation, which is also a direction that we will explore later.

## Data Availability

No datasets were generated or analyzed during the current study.
